# Technological interventions in improving the functionality of proteins during processing of meat analogs

**DOI:** 10.3389/fnut.2022.1044024

**Published:** 2022-12-19

**Authors:** Pavan Kumar, Neelesh Sharma, Muideen Adewale Ahmed, Akhilesh K. Verma, Pramila Umaraw, Nitin Mehta, Ahmed Abubakar Abubakar, Muhammad Nizam Hayat, Ubedullah Kaka, Sung-Jin Lee, Awis Qurni Sazili

**Affiliations:** ^1^Institute of Tropical Agriculture and Food Security, Universiti Putra Malaysia (UPM), Seri Kembangan, Malaysia; ^2^Department of Livestock Products Technology, College of Veterinary Science, Guru Angad Dev Veterinary and Animal Sciences University, Ludhiana, India; ^3^Division of Veterinary Medicine, Faculty of Veterinary Sciences and Animal Husbandry, Sher-e-Kashmir University of Agricultural Sciences and Technology of Jammu, Jammu, India; ^4^Department of Livestock Products Technology, College of Veterinary and Animal Sciences, Sardar Vallabhbhai Patel University of Agriculture and Technology, Meerut, India; ^5^Department of Animal Science, Faculty of Agriculture, Universiti Putra Malaysia, Seri Kembangan, Malaysia; ^6^Department of Companion Animal Medicine and Surgery, Faculty of Veterinary Medicine, Universiti Putra Malaysia, Seri Kembangan, Malaysia; ^7^Department of Applied Animal Science, College of Animal Life Sciences, Kangwon National University, Chuncheon-si, South Korea; ^8^Halal Products Research Institute, Putra Infoport, Universiti Putra Malaysia, Seri Kembangan, Malaysia

**Keywords:** non-traditional proteins, extrusion, electrospinning, couette shear cell, bioprinting

## Abstract

Meat analogs have opened a new horizon of opportunities for developing a sustainable alternative for meat and meat products. Proteins are an integral part of meat analogs and their functionalities have been extensively studied to mimic meat-like appearance and texture. Proteins have a vital role in imparting texture, nutritive value, and organoleptic attributes to meat analogs. Processing of suitable proteins from vegetable, mycoproteins, algal, and single-cell protein sources remains a challenge and several technological interventions ranging from the isolation of proteins to the processing of products are required. The present paper reviews and discusses in detail various proteins (soy proteins, wheat gluten, zein, algal proteins, mycoproteins, pulses, potato, oilseeds, pseudo-cereals, and grass) and their suitability for meat analog production. The review also discusses other associated aspects such as processing interventions that can be adapted to improve the functional and textural attributes of proteins in the processing of meat analogs (extrusion, spinning, Couette shear cell, additive manufacturing/3D printing, and freeze structuring). ‘

## 1 Introduction

During the last two decades, the plant-based meat analogs sector has recorded tremendous growth, attributed mainly to their inherent merits associated with sustainability prospects, nutritive quality, improved food safety, food availability, ethical/animal welfare issues associated with their production process as compared to conventional meat and increasing trend of vegetarianisms (1–5). The EAT-Lancet commission also recommended for reduction in animal-source protein and dairy products intake with a more health diet comprising plant-based proteins such as legumes, nuts, and beans (6) for the betterment of the environment and public health (7). Meat analogs with organoleptic attributes similar to meat and can be promising alternatives for high-quality protein diets of the future with existing, already strained natural resources (8, 9). Animal production utilizes about 83% of the total farmland and make up to 56-58% of total emission but only cater to 37% of total protein and 18% of total calories globally (10).

The increasing numbers of start-ups and food processing companies are focusing on producing higher variations and varieties of meat alternatives by introducing increasing numbers of plant-based analogs for sale, such as burger patties, sausages, and minced meat alternatives. According to an estimate by OECD (11), the plant-based meat analogs had an estimated market value of 4.8 billion USD in 2019 which is expected to reach 6.8 billion USD by the year 2025 with a 7.1% growth rate. Worldwide, Europe has the largest market share of above fifty percent for plant-based meat analogs (51.5%), followed by North America with a share of 26.8%, and Asia Pacific (11.8%). About 6.3% market is in Latin America and 3.6% is in the Middle East and Africa (11, 12).

Meat and meat products are excellent source of highly bioavailable animal proteins and important minerals and have relatively higher digestibility and biological and nutritive values. However, meat and meat products generally have lower storage stability and require efforts in terms of preservation (13, 14) and are often associated with negative consumer perception mainly due to health issues related to the consumption of red and processed meat (9, 15–17) and animal welfare or ethical issues during livestock rearing and slaughtering (15). The association of meat consumption with certain diseases and animal welfare issues has led to an impetus for the development of protein-rich alternatives such as meat analogs and cell-based products (18).

Vegetable proteins form a crucial component of plant-based meat analog production. The carbon and water footprints of these vegetable proteins are significantly lower than those of animal proteins obtained from the slaughter of animals which further has animal welfare, ethical and religious issues. Blue water refers to the volume of fresh water evaporated from the global blue water resources (surface water and ground water) (19). The carbon footprint (g CO_2_e/kg protein), blue water footprint (L water/kg protein), and land-use footprint (m^2^ year/kg protein) of raw ingredients of vegetable origin, such as isolated soy protein are about 2-6, 38-205 and 6-8 respectively, that are much lower than that of beef production, 174-184, 1,607 and 1,310-1,311 values, respectively (20). In addition to the environmental merits, plant proteins are more economic/cheaper and can be processed into various meat alternatives with desirable nutritional and organoleptic properties (5).

This has necessitated the accurate selection of proteins or their suitable combinations as a paramount focus area for production of meat analogs/alternatives. The quality and functionality of plant protein have a critical role in the functional, nutritional and structural attributes of meat analogs (4).

## 2 Protein sources

Proteins from a wide range of sources such as soy, wheat, rice, legume, mushroom, pseudocereals, leaves, oilseeds, fungi and single-cell proteins (SCP) are used for the development of meat analogs. Recently there has been escalated focus on the utilization of protein sources from single-cell protein (SCP)/edible unicellular microorganisms/microbial protein (21), duckweed, seaweed (22), oilseed proteins and oilseed co-products (23), pseudocereals such as Amaranth and Quinoa grains (24), cauliflower, eggplant, jackfruits for processing of meat analogs (25). These have a high potential for sustainability, nutritive value (higher protein content, sulfur-containing amino acids), and price advantages (26, 27) which can be utilized after suitable technological interventions. Leaves from Alfalfa, grass, sugarcane, the mulberry bush, and tobacco has been extracted for their potential application in the development of plant-based meat analogs (27). Other proteins of good potential having good protein solubility in alkaline medium, gelling, foaming, and water absorption are chia seed, pumpkin seeds, hemp, and potato proteins and can also be used in the preparation of plant-based meat analogs (28).

### 2.1 Legume proteins

Soybean due to its cheapness, easy availability, nutritive value (protein digested corrected amino acid (PDCAA) score of 1.0; biological value comparable to conventional meat), functional (emulsifier, chewability, texture, stabilizers, water retention), satiating properties, and organoleptic attributes, makes it the single largest source of vegetable protein used in the preparation of meat analogs (29). Texturized soy protein (TSP) also known as texturized vegetable protein (TVP), or soy meat has 50% protein content which decreases upon rehydration. It is widely accepted as a meat replacer by vegetarians and health-conscious consumers (30). Products like tofu, tempeh, and yuba have become quite popular these days (31). Glycinin, β- and γ-conglycinin, 7S-globulin, lectins, lipoxygenase, and β-amylase are major soy proteins. Soy leghemoglobin, a heme pigment is now increasingly being used for meat analogs preparation for imparting a ‘bloody appearance’ of meat heme proteins (32). Food structure and thus, the texture is largely governed by proteins, polysaccharides, and/or their interactions. Stanojevic et al. (33) reported that the incorporation of inulin carbohydrates in tofu improved its hardness and gel strength through the hydrophobic interaction of soy saponin with carbohydrates. This interaction affected the foam-forming ability and altered its elastic behavior.

Legumes such as pea, lentils, mung beans, chickpeas, and lupin beans are also gaining importance in meat analog preparations. These legumes have poor digestibility compared to soy proteins, but with suitable technological interventions, these proteins might offer complementary desired structural and processing characteristics for developing meat analogs (34). Legume/pulse proteins (beans, peas, lentils, and chickpeas) are increasingly being used to produce gluten-free, low-allergen vegetable protein-based meat analogs with improved textural, nutritional, and organoleptic attributes (35). The legume seeds contain higher protein contents (20-35%) than other plant proteins, are a rich source of dietary fiber, vitamins, minerals, and are low in saturated fats (36).

Pulse proteins have a better amino acid profile than cereal proteins and have a comparable concentration of lysine, leucine, and phenylalanine to soy protein. Lupin beans are rich in protein content (46%) and had the least undesirable non-nutrients (23). Other less common legumes such as chickpea, faba bean, and mung bean proteins are also used for the development of meat analogs (34). Buhler et al. (37) observed the improved water-holding capacity and protein solubility of faba bean protein concentrate; having similar attributes to the soy protein concentrate. Chickpea, a commonly consumed legume has a positive effect on color (similar to meat due to the presence of carotenoids) and good textural and water holding and oil binding properties (38). Chickpea protein isolates had higher water and oil-binding properties than soy protein isolates (39).

Globulins form the major storage proteins in pulse seeds (70-80%). The major fractions of globulin are legumin (11S) and vicilin (7S) with convicilin as a minor fraction (35). Comparatively lower gelling strength of pea proteins (except mung bean and chickpea) could be overcome by suitable processing technology such as the incorporation of hydrocolloids or polysaccharides, less refined protein-rich ingredients, ultrasonication, starch enrichment, dehulling, dry fractionation, milling, air classification, alcohol (ethanol or isopropanol) washings (40, 41).

Zhu et al. (42) explored the processing of pea protein and its effect on the functionality suitability for the production of meat analogs developed from such proteins. Two separation processes, namely dry separation which consisted of ultrafine milling, air pre-classification, and electromagnetic separation, and wet separation done by isoelectric point precipitation were employed to obtain pea proteins. A marked variation in the purity of pea proteins was obtained by dry separation (72.3 g/100 g) and by wet separation (89.2 g/100 g) (42). Functional properties like emulsification and foamability were better in pea proteins obtained by the wet process while water absorption was superior in dry-separated pea protein isolates. Authors noted better natural structure, and solubility of pea protein isolates obtained by the dry method, formulating porous and soft meat analogs. In contrast, pea protein isolates obtained by the wet method had better amino acid content near to the recommended level. These pea protein isolates as well as wheat gluten have great potential in the development of meat analogs (43).

The interfacial and rheological properties of pea protein isolates were improved by soy soluble polysaccharides (SSPS) which facilitated the rearrangement and reconnection of modified pea protein particles. SSPS consists of a galacturonan backbone of homogalacturonan (a-1,4-galacturonan) and rhamnogalacturonan (repeating units of a-1,2-rhamnose and a-1,4-galacturonic acid) branched by b-1,4-galactan and a-1,5-arabinan chains (44). SSPS has a pectin-like structure and emulsifying property due to the presence of glycoproteins (45). Zhan et al. (45) reported a significant reduction in hydrophobicity and increased emulsion stability of pea protein isolates by adding freeze-dried soy soluble polysaccharides (SSPS). The functionality of pulse proteins can also be improved or modified by technological interventions such as methods of extraction. Protein-rich faba bean flour prepared by dry-fractionation was reported to exhibit superior functionality, protein solubility (85% at pH 7), foaming capacity, and gelling ability compared to isolate produced through isoelectric precipitation or acid extraction (46).

Similarly, Malik and Saini (47) observed improvement in emulsifying, foaming properties, and oil binding capacity of sunflower protein isolates by heat processing at pH 4-5 due to minimum denaturation of proteins at this pH. Not only the purified proteins but even their partially fractioned components contribute to their functional properties. The partially fractioned ingredients of sunflower such as polysaccharides, oil macromolecules as well as proteins exhibited good emulsifying potential comparable to commercially used proteins (48).

The application of ultrasound technology has also been explored for improving the functionality of proteins as high-intensity ultrasound treatment changes protein isolates’ secondary and tertiary structures, thus altering its gel strength. The ultrasonication at below 10°C for 5-30 min (probe-20 kHz, 25% amplitude with power output-500 W and ultrasonic intensity-58-61 W/cm^2^; probe-40 kHz with power output-500 W and ultrasonic intensity-0.5 W/cm^2^) of sunflower isolates resulted in the unfolding of proteins, exposing its hydrophobic groups leading to non-covalent interactions between denatured protein molecules (49). These non-covalent interactions reinforce the colloidal network during cooling (49) and promote a meat-like texture.

Rapeseed proteins have good foaming, emulsification, and gelation properties, and thus are being increasingly utilized in plant-based sausage analogs. Transglutaminase, polysaccharides like gum arabica, and chemical modifications such as acetylation or succinylation have been successfully employed to increase the gelation of rapeseed (canola) proteins (50). Li et al. (50) studied the effect of gum Arabic and pH on rapeseed protein isolate emulsion (3% w/v) and observed a stable emulsion with a particle size of 314 nm and absolute zeta potential value of −44.3 ± 0.5 mV at 1% gum Arabic and at pH 8. Authors (50) attributed it to a significant increase in β- sheet and a decrease in β-antiparallel due to the interaction between rapeseed protein isolates and gum Arabic mainly via changes in hydrogen bonds.

### 2.2 Wheat gluten

Wheat gluten, a byproduct of the wheat starch industry, owing to its visco-elastic properties, is commonly used as traditional cereal protein for preparing meat analogs for providing structural and fibrous attributes. Wheat gluten has excellent binding properties, baking performance, viscosity, swelling, leavening properties, and dough preparation attributes due to the formation of viscoelastic polymeric networks capable of retaining gases in it (51). It improves the nutritional quality of meat analogs (a rich source of glutamine) and is used as a binder and structural agent. Moreover, the structural characteristics and cross-linking in meat analogs are also affected by various processing conditions such as isolation of specific proteins, protein modification, pressure, temperature, chemical pretreatment, or presence of alkali, salts, or polyphenols (52–54). The incidences of gluten allergy and celiac diseases have forced food technologists to search for a suitable replacement for wheat gluten by modifying properties of other proteins similar to wheat gluten and cross-linking by fermentation, hydrolysis, or application of transglutaminase, phenolic compounds, and organosulfur compounds (55–57).

### 2.3 Zein

Zein is a major storage protein in maize. It forms viscoelastic networks above its glass transition temperature (the temperature at which a hard glassy structure change to a rubbery state). Its inability to be drawn into long fibers or sheets at room temperature is the greatest hurdle for food technologists to employ zein in the preparation of meat analogs (58). Mattice and Marangoni (59) developed thin strips or ribbons of zein by extruding zein-ethanol solutions of which the ethanol was removed later by air drying. The self-assembled zein networks had comparable rheological behavior to chicken breast, thus presenting an opportunity for zein in plant-based food structuring. The intermolecular β-sheet structure has the potential to improve viscoelasticity, hence improving cohesiveness and proper mouthfeel in the developed meat analogs (60). Erickson et al. (60) proposed atomistic modeling of peptide aggregation and β-sheet structuring for improving the viscoelasticity of corn zein. The authors observed that peptide sequences without proline had a higher level of β-sheet structuring.

The β-sheet structure, visco-elastic dough with improved mixing stability was developed with rice flour and zein (above 5% w/w level) for the development of gluten-free dough sheets capable of forming long noodle strands (58). Zein, although lacking certain essential amino acids, has great potential in meat analog developments due to its fibrous nature. Further, the enzymatic and chemical alterations of zein, such as hydrolysis, deamidation, and cross-linking, could be very useful tools in improving the functionality of zein protein for its wider application in the preparation of meat analogs (61).

### 2.4 Rice protein

Recently rice protein is gaining importance in the development of gluten-free meat analogs. As compared to soy protein, rice protein has a milder flavor and bland taste. Further rice proteins are also having hypoallergic and hypocholesterolemic effects (62). Rice protein consists of albumin (30.9%), globulin (24.9%), glutenin (32.5%), and prolamin (11.6%) (63). It is a rich source of arginine, cysteine, histidine, methionine, and valine amino acids. The lysine (3.8%) and leucine (8.2%) amino acid content are higher than wheat (64). Rice protein had higher nutritional quality, true digestibility, and biological value as compared to soy protein isolates (65), hence recommended for infants and the elderly (58). Rice bran is a rich source of dietary fiber (27.6-33.3%) and its incorporation in meat analogs could improve the nutritional as well as functional properties of the developed meat analogs (66, 67).

The full replacement of rice protein during the extrusion is challenging due to its low porosity (68). The incorporation of isolated rice protein (10%) along with isolated soy protein (40%) and wheat gluten (40%) and corn protein (10%) was observed to improve the textural properties (integrity index, chewiness, and degree of texturization) and decrease the formation of the fibrous structure upon extrusion. The raw materials were extruded at 140°C barrel temperature, 250 rpm screw speed, 100g/min feed rate, and 40% moisture. Xiao et al. (67) noted enhanced intermolecular hydrogen bonds leading to increased hardness and tensile force at temperature (<170°C) and increasing screw speed up to 280 r/min during the extrusion of rice bran-added meat analogs. Increasing moisture content was reported to a lower hardness and tensile force values during the extrusion of rice bran-added meat analogs (67). Red yeast rice, prepared by culturing rice with *Monascus purpureus* yeast strains, improve the color, high moisture, protein, and fat content but with significantly (p < 0.05) decreased shrinkage and cooking yield of the plant-based meat analogs as compared to beef patties (69).

### 2.5 Mycoproteins

Mycoproteins obtained from edible fungi such as obtained from *Fusarium graminearum* is gaining popularity in the development of novel meat analogs due to their comparable nutritive value to meat proteins (such as fiber source, polyunsaturated fatty acids, minerals, vitamins), texture (formation of flaky or fibrous texture) sensory attributes (meaty flavor, taste), sustainability and high digestibility, which can be further improved by using egg albumen proteins (4). Mycoproteins are considered a sustainable source of complete nutrition (essential amino acids, vitamins, carotene, minerals, and carbohydrates) with 10-20 times less land requirement and 10 times lower carbon emission than beef production, and 4 times lower carbon emission than chicken production (70). Mycoprotein has a biological value similar to milk proteins, and on average, 100 g of dry matter of mycoprotein contains 45% protein, 13% fat, 10% carbohydrates, and 25% fiber on average (71). As compared to other common plant proteins, mycoprotein contains a higher weight-percentage protein content. The fiber present in fungal cell walls is mostly made up of β-glucan (up to 75%) and chitin; consequently, forming a ‘fibrous chitin-glucan matrix’ (71). The mycoprotein is classified under the food category ‘high in fiber food’ by the European Commission (72).

### 2.6 Mushroom protein

With proper incorporation of gelling and thickening agents, good-quality meat analogs can be prepared by using mushrooms as a source of protein and nutrition. Arora et al. (73) prepared good quality mushroom-based sausage analogs by using carrageenan and xantham gum as binding agents and reported significantly improved textural attributes, emulsion stability, and decreased purge loss upon the incorporation of 0.8% carrageenan concentration. Kumar et al. (74) developed analog meat nuggets by incorporating mushrooms into texturized soy protein and wheat gluten (74–76).

### 2.7 Pseudocereals

Quinoa seeds, categorized under pseudocereals can also be used as a binder or extender replacers in place of texturized vegetable proteins during the preparation of meat products for improving binding, water retention, and cooking yield. Quinoa protein exhibit emulsifying, foaming, and water absorption properties similar to soy protein, and shows good gelation at a low pH in the presence of divalent salt addition (77).

### 2.8 Potato protein

The potato processing industry generates a huge amount of potato proteins that can be utilized to improve the functionality of non-functional plant proteins such as zein fractions. At present, a lot of potato protein goes to waste and their proper utilization in food is considered a sustainable and economically beneficial option (78). Commercial preparations such as Quorn^®^ vegan nuggets, Gardien-seven-grain crispy tender, Beyond meat-sausages, and Impossible foods-impossible burgers are made with potato proteins as an ingredient (79). Potato proteins have good emulsifying capacity, foaming, and gelation properties. Patatin is the major potato protein that undergoes denaturation (destruction of tertiary structure and forming random polypeptide chain) at a lower temperature (55-75°C) and forms a good gel network (80). Glusac et al. (81) obtained a stable emulsion with potato proteins and zein which remained stable for 30 days. The oil-in-water type emulsion was stabilized by the potato proteins solubilized in the aqueous phase with the help of cross-link formed by enzymatic action by tyrosinase enzyme obtained from *Bacillus megaterium* and zein solubilized in the oil phase. The enzymatic-assisted cross-link formation between potato protein-zein resulted in 10-fold larger particle size in the form of covalent aggregates, 40-fold higher viscosity, and a 2-fold increase in hardness value as compared to control emulsion and has the potential to be used in meat analogs preparations (81). For improving nutritive value such as dietary fiber, antioxidants, vitamins, and ash contents, a suitable combination of raw ingredients is recommended (82).

### 2.9 Single-cell protein

Single-cell protein is harvested from microalgae, bacteria, and fungi cultivated on food-grade substrate, beverages, or food industry wastes by solid-state or submerged fermentation (8). Algae can be grown on a wide pH range (up to 11) whereas bacteria and yeast grow at a slightly neutral to acidic medium (pH 5-7). To harness the vast potential of protein from aquatic sources (seaweeds, and single-cell-protein), the proper extraction technology for their separation and purification is needed (83). The high content of nucleic acid (up to 16% dry weight in bacterial cells) in SCP is associated with their rapid growth. The nucleic acid content in SCP should be controlled as these nucleic acid metabolized into uric acid in the human body, thus increasing the risk of kidney stones and gout (84). It can be decreased by suitable alteration of growing conditions, and proper extraction protocols such as endogenous RNAase, alkaline hydrolysis, chemical treatment, and autolysis.

Commonly used major proteins used in the preparation of meat analogs are presented in Table 1.

**TABLE 1:** Characteristics of major proteins used in the preparation of meat analogs.

Source	Vegetable protein	Composition (% dry matter)	Functionality	References
Soy	Soy flour	43-56% protein, 3-7% crude fibers	Water binding capacity, oil-absorbing capacity, emulsifying capacity	(5) @A
	Texturized soy protein/Soy meat/Soy protein concentrate	70% protein	Texturizing attributes, protein source	
	Soy protein isolates	90% protein	Gelling, WHC, solubility lower upon heat treatment	
Wheat	Wheat gluten/Wheat meat	25% protein	Dough-making ability, baking ability, binding, texture	(85) @A
	Wheat gluten isolates (glutenin, gliadin)	75-80% protein, 15-17% carbohydrates	Binding, dough making, disulfide cross-link	
Rice	Rice flour, defatted flour	6-10% protein	Cooking and eating quality	(26, 27) @A
Pea	Pea protein isolate (pea globulin, albumin)	85% protein	WHC, gelling, emulsifying, air incorporating	
Groundnut	Defatted flour	44-50% protein	Emulsifying activity and stability, foaming capacity, good water binding and solubility	
Sesame	Sesame seed, defatted flour	19-27% protein, 2.5-4% dietary fiber	Texture, color, organoleptic attributes, emulsifying and foaming	
Corn	Zein	8-11% protein	WHC, poor gelation, emulsifying, foaming activity	
Sunflower	Deoiled sunflower seed meal, sunflower protein concentrate	20% protein in deoiled seed meal	Protein source, emulsifying ability, moderate WHC similar to SPC, thickening agent	
**Single-cell protein (SCP)/Petroprotein**				
Microalgae	*Chlorella*, *Scenedesmus*, *Spirulina*	50-80% protein	Emulsifying, foaming, water and oil binding capacities, 4-6% nucleic acid, more prone for contamination and accumulation of toxic compounds and heavy metals	(86) @A
Fungi	*Candida*, *Torulopsis*, *Saccharomyces*, *Aspergillus niger, Sporotrichum pulverunetum, Fusarium graminearum, Penicillium cyclopium*,	Essential amino acids profile similar to soybean oil meal	High amount of nucleic acid (up to 10%), texture, foaming capacity, foaming stability and emulsifying property	(70) @A
Bacteria	*Cellulomonas* and *Alcaligenes*	80% Protein methionine (2.2-3.0%)	A total of 15-16% nucleic acid, a rich source of protein having high biological value, essential amino acids	(87) @A

WHC, water holding capacity; SPC, soy protein concentrate; SPI, soy protein isolates.

## 3 Protein extraction

Herbaceous (lucerne, leaves from agro-industrial crops such as cassava, sugarcane, and trees) and aquatic biomass (aquatic plants such as duckweed, seaweed, macro, and microalgae) are explored for ensuring sustainable food supply due to their high protein content, higher yield, efficient protein conversion and availability on large scale (88). Protein extraction mainly focuses on the extraction of soluble proteins from green biomass. In leaves, rubisco (ribulose-1,5-biphosphate carboxylase/oxygenase) forms the major portion of soluble proteins (83). Improving protein extraction yield from comparatively sustainable aquatic biomass (microalgae, seaweeds, aquatic plants) is the present focus of the food industry by exploring and employing a range of technologies such as tissue disruption, protein solubilization and fractionation, protein precipitation and purification and concentration (83).

The protein extraction from microalgae is quite challenging due to the presence of pigments and polysaccharides in protein due to cell disruption, hence requiring a proper purification process. Similarly, during the extraction of protein from seaweed, polysaccharides and phenolics are present as co-extracted compounds and interact with seaweed proteins (89). The microalgae cell wall can be broken by bead milling, high-pressure homogenization, or by a combination of various physical treatments (such as microwave, ultrasonication, autoclaving, grinding, and osmotic shock), enzymatic treatment, or by chemical treatments (58, 90, 91). Protein extraction is performed by solubilization of proteins in an alkaline solution followed by isoelectric precipitation at pH 3-5. Other methods applied for protein extraction are precipitation with solvent, adsorption, and ultrafiltration (89). Depending upon the plant source and extraction process, the leaf protein concentrate form about 40-60% of the total leaf protein (92). The application of enzymatic mixtures degrades the structural components of the cell wall and better releases soluble components containing proteins.

Protein fractionation from seaweeds is a very complicated process due to the presence of phenolic compounds (chemically bind to proteins) and cell wall mucilage/neutral polysaccharides (reduce mass transfer). The seaweed protein extraction is done by drying the biomass followed by alkaline extraction, enzymic digestion, osmotic shock, or high shear grinding (93). However, the processing of fresh seaweeds was observed to improve extraction yield, increase protein concentration, and higher total essential amino acids (94).

The functionality of a protein depends upon the extraction protocol such as exposure to high temperature or high alkaline pH could denature proteins, consequently reducing their functionality. Non-hydrolyzed or partially hydrolyzed large proteins are preferred for gelling and textural functions (95), whereas hydrolyzed small proteins have good emulsifying properties (96). An increase in protein functionality has also been observed in the presence of impurities such as charged sugars (97).

The protein recovery methods have a direct impact on the functionality and quality of plant protein such as albumins retention in pea protein obtained by ultrafiltration and dialysis, whereas globulins retention under alkaline-isoelectric precipitation and micellar precipitation (98). Alkaline extraction also significantly impacts pea protein configuration by exposing hydrophobic regions with higher gel strength, whereas salt-dialysis prevents protein folding leading to a coarser structure and soft gel (98). Pea protein concentrate forms a fine elastic gel with albumin fraction F forming a weak gel due to low purity, whereas globulin-rich fraction forms a fine elastic gel. The globulin-rich fraction and albumin fraction of pea protein might be used as an alternative to whey protein isolates (99).

**FIGURE 1:**
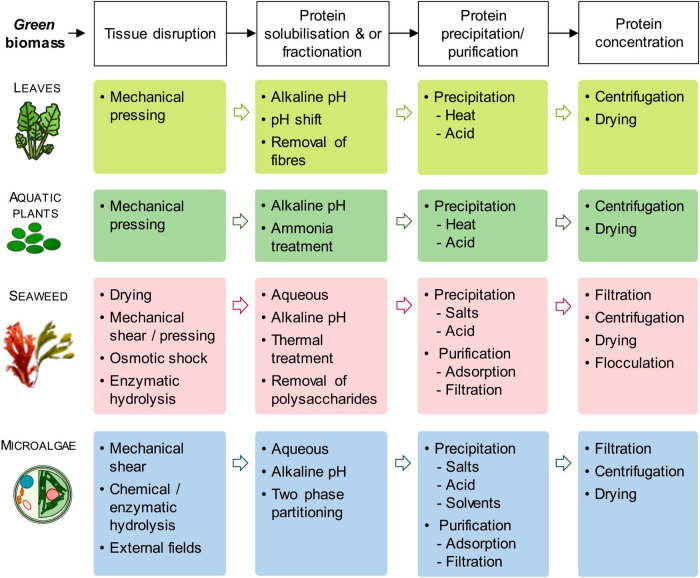
Generic protein extraction process from green biomass (top line) and an overview of extraction methods for each biomass group. [Adopted from Tenorio et al. (83)] under Creative Commons CC-BY-NC-ND license).

Figure 1 summarized the various protein extraction process.

The protein separation is based on the variations in structural, functional, or chemical properties between the target compound/protein and other proteins in the mixture. These variations can be in size, shape, molecular weight, density, specific gravity, charge, solubility, charge distribution, metal binding, ligand binding, posttranslational modifications, and specific structures or sequences (100). Membrane technologies viz., microfiltration, ultrafiltration and nanotechnologies are increasingly used as energy-efficient and green technologies for the separation and purification of bioactive compounds (101, 102).

Tags are specific fusion peptides or protein sequences that are used to improve the efficacy of the protein purification process by determining interactions between proteins and immobilized ligands (103). Tags can be a whole enzyme, a small polypeptide chain or a protein domain and interacts with a range of substrates such as metal chelators, carbohydrates, antibodies, and small molecules, thus enhancing the yield, purity, and recovery of associated counterparts (104). Further tag-assisted protein purification can have potential to produce purified proteins at commercial scale if the issues of design and application of tagged fusion proteins are properly addressed (105). For a successful tagging protocol, it should provide end protein products with high yield, purity and should preserve their natural structure and functionality.

The application of nano-sized resins in chromatography column has resulted in a significant increase in process rate, resolution, and purity of final products. It reduces the time of the experiments and increases mass transfer, decreases the loading capacity of the column, and increases the surface-to-volume ratio and load of the affinity ligands (105). Further with nanofabrication, the mechanical strength of resins can be significantly improved, consequently reducing the total cost of separation or purification of desired compounds (106, 107). Various methods of protein purification are summarized in Table 2.

**TABLE 2:** Various methods of protein purification.

Process	Principle	Medium	Remark	References
**Low-resolution methods**				
Precipitation	Solubility	Ammonium sulfate	Salting-in, yield can be improved by optimization of ammonium sulfate concentration,	(108) @A
		Organic solvents	70-75% concentration of organic solvents leads to precipitation, loss in solubility	(109) @A
		Polyethylene glycol	First step of purification by precipitating proteins	(100) @A
	Solubility and isoelectric	Isoelectric point	Net charge of protein becomes zero at isoelectric point, reduction of repulsive electrostatic force leading to aggregation and precipitation	(109) @A
	Molecular identification, solubility	Reversible soluble-insoluble polymers (affinity precipitation)	Improve purification efficiency by reducing volume at initial steps of downstream processing	(110) @A
	Charge and size	Polyethyleneimine (PEI)	Salt dependent, protein recovered from PEI-protein complex by elution with salt	(111) @A
**Low-medium resolution methods**				
Phase partitioning	Solubility/Hydrophobicity	Aqueous two-phase partitioning	Liquid-liquid fractionation method, partition behavior difficult to predict, high yield, easy scale-up	(112) @A
		Three-phase partitioning	Efficient, fast, safe and green method, comprises three steps viz., salting out, isoelectric point precipitation followed by solvent precipitation	(113) @A
**High-resolution methods**				
Chromatography	Gel filtration/Size exclusion	Molecular size	Versatile, commercially available matrices, mild operational protocol, low ratio of sample volume to column ratio	(114) @A
	Ion exchange	Surface charge	Use of suitable media as per proteins isoelectric point, separating negatively or positively charged molecules via their interactions with stationary media of charged ion exchange resin	(115) @A
	Hydrophobic interaction	Hydrophobicity	Used to remove various impurities in solution mainly product aggregates possessing different hydrophobic properties	(116) @A
	Affinity chromatography	Affinity of proteins for specific molecules	Efficacy, high level of resolution and purity, purification of recombinant and native proteins	(117) @A
	Reverse-phase HPLC	Size, hydrophobicity	Method of choice for peptide separation, easier scale-up, suitable for mass spectrophotometry	(118) @A
	Perfusion chromatography	Two sets of perfusion media, through pores (6000-8000 A) and diffusive pores (800-1500 A)	High speed, low cost, loading capacity not depends upon flow rate, convective and diffusive flow makes better accessibility of inner particles to macromolecules	(119) @A
	Tags assisted chromatography	Ligand binding, selective and specific interaction	High specificity, high yield, selective and specific affinity, specific tags for specific proteins	(105) @A
Nanosized resins	Gold nanoparticle	Ligand binding, specific interactions	Increased mass transfer, purity and separation, user-friendly, affordability, selectivity	(120, 121) @A
	Carbon nanotubes	Ligand affinity, specific adsorption	Improved surface: volume ratio, increase load of affinity ligands, wide application in pharmaceutical industry	(122, 123) @A
	Mesoporous silica	2-50 nm pore size, fast flow, rapid separation	Ease of operation, durability, inert materials, low toxicity, large surface area	(124) @A

## 4 Improving functionality of plant proteins

Protein ingredients used in the production of meat analogs are among the essential aspects for product characteristics and differentiation. Essential aspects defining the structure and function relationship of protein include hydration and solubility properties, emulsification and foaming properties, flavor binding, viscosity, gelation, texturization, and dough formation (125). The functionality of proteins, on the other hand, maybe further altered by processing, resulting in new conceivable uses for the proteins (126). Other factors to consider include protein cultivar type, extraction methods, and drying methods, which influence the functional ability of plant protein ingredients (127). The fractions of albumin and globulin in plant proteins vary with cultivars and genotypes and various cultivars and genotypes have different components; thus, demonstrating variations in their functional properties (128). For example, water-soluble fraction of red lentil Firat and green lentil Pul II was reported to vary significantly, with red lentil protein concentrate had a higher water-solubility as compared to their green lentil counterparts (129, 130). Other functional parameters observed in lentils to varies with cultivars were gel-forming ability, oil absorption capacity, foaming stability, and foaming capacity (131). The protein concentration, molecular conformations of different types of proteins, types, and aggregation state play an important role in determining its functionality. The presence of other ingredients in proteins such as fibers, polysaccharides, lipids and minerals have impact on functionality (127, 132).

The water-holding capacity of protein isolates is usually greater than water-holding capacity than their corresponding flour. The presence of some non-protein ingredients such as fibers, lipids, and starch granules act as barriers to water penetration such lentil protein isolates demonstrated higher water-holding capacity than lentil flours, mainly attributed to lower lipid content and smaller particle size (133). Similar is the case with chickpea protein isolates had higher oil holding capacity (2.1-4.0 g/g) as compared to their corresponding flour (1.1-1.2 g/g) (134).

The functionality of plant proteins could be significantly modified by the application of suitable processing technologies. The solubility of proteins can be modified by heat, acid- or alkali-induced denaturation, hydrolysis, and heat-labile proteins can be enzymatically hydrolyzed to improve their solubility, heat stability and lowering allergenicity (135, 136), The proteins harvested by processing entire organisms such as algal proteins, mycoproteins, and insect proteins show a high level of heterogenicity (137). During extraction and processing, plant proteins are often undergoing severe heat, shear, or solvent extraction processes. These processes result in denatured and cross-linking or even hydrolysis of proteins. The functional and nutritional properties of oilseed proteins is affected by the extraction conditions (138).

Heat-induced denaturation of proteins leads to their aggregation via hydrophobic interactions, hydrogen bonds, and disulfide bonds. Heating of proteins at low or high from isoelectric pH or low ionic strength results in the filamentous structure of a fine-stranded aggregates with a string-of-beads shaped structure. Further, this aggregation becomes more random at moderate or high ionic strengths resulting in particulate aggregates (139, 140). With the addition of salt, the filamentous aggregates form physical entanglement networks, thus masking electrostatic repulsion. Hydrophobic interactions induce network formation in solutions of filamentous aggregates and on their gel formation, other forces also consolidate the network, resulting in further improving gel strength (139), homogenous cold-set gels are transparent (140). Particulate or heterogeneous gels have a turbid appearance and low water-holding capacity (141).

Globular proteins have non-polar regions on their surface which improve adsorption to oil-water or air-water interfaces, consequently improving emulsifying and foaming potential. Albumins from peas and chickpeas have lower flexibility and hydrophobicity leading to overall lower emulsifying properties (142, 143). The albumins of kidney beans exhibit good foamability and emulsifying properties resulting in their good solubility, lower molecular weight, and better molecular flexibility, however, these kidney beans are unable to form a stable foam at neutral pH as compared to globulins (144). Under optimum ionic strength and heating conditions during food processing, the individual units of 7S and 11S globulins undergo disaggregation-unfolding-reaggregation resulting in improving their functionality viz., solubility, gelation, emulsification, and foaming (145).

There are some challenges in the utilization of plant proteins such as allergenicity to some peoples, off-flavor, presence of antinutrients (such as glucosinolates in rapeseeds) which can be ameliorated by proper selection and incorporation levels, implementing suitable extraction protocols (cold-pressed, microwave-assisted), use of a proper solvent such as ethanol, microbial fermentation, plant-breeding and other processing technologies (146–149). The plant functionality could be improved by proper technological interventions.

### 4.1 High-pressure processing

High-pressure processing (HPP) reduces the functionality of plant proteins reducing particle size due to cavitation and fragmentation of macromolecules. The reduction in particle size was observed in hazelnut, and lentil proteins upon high-pressure processing up to 150 MPa (150). Over-pressing above 150 MPa results in a decrease in protein solubility such as lentil and hazelnut proteins (151). The appropriate pressure depends upon the protein types and solvent, with a pressure drop leading to an increase in particle size due to the denaturation of proteins under mechanical forces while dropping the pressure and higher temperature at the homogenizer valve (152). The particle size reduction of proteins leads to higher emulsifying and foaming properties owing to rapid movements of proteins toward the air-water interface whereas decreased foaming properties could be due to the lower intermolecular interaction and higher unfolding of proteins.

### 4.2 Extrusion

The high temperature applied during extrusion results in the disintegration of hydrogen bonds, thus the unfolding of proteins. The stabilization of extended protein networks is stabilized by increased protein aggregation formed by α-helices, β-sheet, non-covalently bonded β-turn or anti-parallel β-sheet structures via breaking of intermolecular disulfide bonds and formation of new intermolecular bonds (153, 154). Further, high temperature and mechanical pressure also destroy the anti-nutrients, thereby improving the digestibility of plant proteins and availability of amino acid. Chickpea flour had the highest PDCAAS (protein digestibility corrected amino acid score) and protein digestibility upon extrusion as compared to cooking or baking process (2, 155). Similarly higher *in vitro* digestibility of extruded soybean protein concentrate was reported by (156) as compared to uncooked samples.

### 4.3 Sonication

Sonication also modifies the functionality of plant protein functionality via localized hydrodynamic shearing of the native protein particles due to cavitation, microjet and violent agitation effect, and thermal degradation due to exposure to high temperature (157). Sonication of protein aggregates was observed to break into smaller particles and a better uniform distribution under the exposure to high-intensity ultrasound effect (158). Similar findings were also reported by Xiong et al. (159) upon sonication of pea protein isolates (5% w/v) solution at 30, 60, and 90% at 20kHz for 30 min. This particle size reduction upon sonication leads to a lower in intermolecular association which further increases more sulfhydryl (SH) groups and hydrophobic regions to be exposed (159). The reduction in the size of the protein aggregates due to the breaking of non-covalent interactions is accompanied by increasing its solubility (2). The increased protein solubility was reported for 1% w/w wheat and soy protein isolates solution (20 kHz frequency, 95% amplitude, 2 min) and 0.5% w/v walnut protein isolate (200, 400, and 600 W for 15 and 30 min) (160).

However, in some cases, sonication with long duration and low power or intense sonication was observed to form larger aggregates of proteins (160) such as 5-fold increase in size of buckwheat protein isolates upon sonication at 100% amplitude for 10 min (161). This could be due to the increase in particle sizes of protein aggregates due to the disruption of microstructure of protein due to sonication leading to swelling of the protein particles in an aqueous medium or self-assembly of unfolded regions of proteins via hydrophobic interactions (161). The authors also observed an increase in reactive content and sulfhydryl (SH) groups, with lower disulfide (SS) bonds due to exposure of SH contents buried inside and breaking SS bonds due to cavitation effects under sonication. Overall, the sonication of buckwheat proteins was observed to alter secondary structure resulting in improved digestibility. Sonication also induces structural changes in plant proteins due to the disintegration of non-covalent bonds, consequently disrupting the secondary structure and partially denaturing the tertiary and quaternary structure without having any significant impact on the primary structure (162).

### 4.4 Chemical modifications

Various chemical modifications by pH change, glycation, and enzymatic action also can induce structural changes in proteins, thus altering their functionality. Derivatization comprises a chemical modification in which reactive side chains in the protein structures are chemically changed to the desired level so to alter their physiochemical attributes and functional characteristics. The amine target groups for derivatization are amino, carboxyl, disulfide, indole, phenolic, imidazole, SH, thioether and guanidine (145). Glycation can be performed chemically by Maillard reaction or by cross-linking enzymes such as laccase and transglutaminase. Maillard reaction is preferred over other chemical methods as it occurs naturally and spontaneously during food processing (163). This non-enzymatic browning occurs via a series of non-enzymatic processes involving the carbonyl groups of reducing carbohydrates and free amino groups of protein. To get the desired yield, sensory, and functional properties of glycated proteins, various factors such as time, temperature, water activity, the concentration of reactants, and pH (164).

## 5 Processing of proteins

Various technologies interventions viz., thermo-extrusion, spinning, and cross-linking are used to impart fibrous texture to meat analogs, and a range of nutrients such as vitamins, flavorings, and minerals are added to make the product equivalent to meat in texture, appearance, and taste. The amino acid sequence is considered a major factor in determining the functionality of plant proteins and can be further improved by applying suitable processing technologies. The nutritional profile of meat analogs can be easily modified to make them healthier (essential amino acids, lower saturated lipids, cholesterol-free) and better for the planet (165). Chiang et al. (166) observed that fibrous meat analogs have the highest content of plant proteins (20-50%), followed by vegetable lipids (up to 5%), starch (2-30%), and miscellaneous minor ingredients. Polymerization of wheat gluten has a direct effect on the product quality during the extrusion and development of meat analogs. Wheat gluten polymerization is affected by thermal and mechanical treatment, with disulfide bonds playing a major role under reducing and non-reducing conditions. Shear rate at 50/sec did not have any perceivable effect on the covalent bond formation (167). Schreuders et al. (168) contemplated the rheological properties of plant protein blends for the preparation of meat analogs with texture maps. Meat analog blends were prepared with pea protein isolates, wheat gluten, and soy protein isolates. In the pea protein-wheat gluten blend, pea protein had a lower strain and elasticity value, whereas soy protein-wheat gluten blends were more elastic and rigid than wheat gluten-pea protein blends (168).

Plant proteins are texturized and processed to make them nutritionally (digestibility, bioavailability), functionally and structurally similar to meat for better acceptance among consumers and their utilization in the preparation of meat analogs at an industrial scale. This step of structuring plant proteins into matrices and their textural and meat-like appearance has a significant effect on the acceptance of meat analogs (169). The texture of plant proteins is affected by the composition and processing conditions. The processing of plant proteins is required to transform these proteins into materials resembling meat in texture, mouthfeel, flavor, and taste (153).

The phase-separation of protein-water systems into water-rich and protein-rich phase is the first step in fiber formation during processing (170). These fibers are further elongated into long strands with the application of shear force, thus leading to an anisotropic and fibrous mass. This phase separation could be further improved by adding a polymeric compound such as carbohydrates or hydrocolloids due to thermodynamic incompatibility as the repulsive forces between various polymers lead to their mutual exclusion (171).

Water has a plasticizer effect on the protein network and affects its rheology (172). During processing, the protein matrix transforms into a set and stable matrix with good mechanical properties needed for meat analog development. The hydrophobic content, cysteine, and charge-bearing amino acids content in a protein affect the stabilization of the protein matrix by forming covalent bonds or by non-covalent interactions (173). The formation of covalent bonds leads to the transition of the protein matrix into a coagulated and thermoset mass.

Various processing technologies used for modifications of vegetable proteins into meat analogs are described in the following sections-

### 5.1 Extrusion cooking

Extrusion technology is used for compressing and reshaping of raw materials by passing this in between dies of desirable shapes and sizes to develop food products with cross-section forms with different shapes and varieties (174). Extrusion is used for texturizing and structuring thermoplastic proteins into anisotropic and fibrous mass for utilization in the production of meat analogs (175). Extrusion cooking consists of a process of continuous mixing, shearing, heating, and forming a mass with one or several screws within a heated barrel. Hot extrusion is done at temperatures above 100°C leading to a cooking effect (structural and chemical changes) depending upon various factors such as particle size, moisture and protein content of raw ingredients, extrusion rate, barrel temperature, and pressure applied.

During the preparation of texturized vegetable protein (TVP) from defatted soy meal, textural attributes of the extrudates contributed to the disulfide bonds, hydrogen bonds, and hydrophobic interactions in the barrel or cooling zone, with more covalent bonds formed under extreme temperature and pressure (176). Upon increasing temperature during the extrusion of texturized vegetable protein from the optimum level (such as 150°C-160°C), a lowered texturization due to protein degradation with small pits appearing on surfaces and brown color was noticed (176). A further increasing temperature above had a detrimental effect on protein, resulting in unstable extrudates.

This technology has great potential in texturing the vegetable proteins by either increasing vegetable protein content or starch content during extrusion cooking as well as by suitable adjustment of processing parameters (system parameters, process parameters, and product parameters), die configuration, pressure, and temperature, screw length to diameter ratio, screw speed, moisture content, and extrusion rate (177). The protein quality and types and pre-treatment of raw materials also have an effect on conformational changes and textural attributes of vegetable proteins. The size constraints of end products, high energy, and high cost of extrusion are some challenges faced by the extrusion industry, and food technologists are working to overcome these challenges. The higher energy consumption in the extrusion process while producing meat analogs may dampen the sustainability merits associated with these products.

The overall quality and texturization are affected by the extrusion parameters (overall effect of shear force, temperature, and pressure), leading to denaturation, aggregation, the association of proteins, lipid oxidation, carbohydrate degradation, and complex conformational changes caused by interactions (molecular conformation, cross-linking) among vegetable protein, lipid, carbohydrate, and other molecules in the extrudate (45). These changes affect the texture, color, and shape of the extrudate. Moisture addition has lubricating and plasticizing effects, decreasing viscosity and the force needed to move material at high barrel temperature resulting in improved texturization of vegetable proteins.

The incorporation of 10% wheat starch into soy protein isolates (SPI) was noted in improving texture extrudates (178). This texturization is attributed to the increasing porous structures, protein network, and increased air cell size. Increased moisture in extrudates has been reported to improve the fibrous structure of vegetable proteins during extrusion. The increased die configuration/diameter was reported to hasten the extrusion process and increased the integrity index (a representative of the degree of texturization more for 8 cm die configuration than 5 cm die configuration), whereas higher water injection rate had a deteriorative effect on it (179).

Li et al. (180) reported a significant increase in gluten polymerization and improved textural attributes of wheat gluten proteins upon alkali treatment. The wheat gluten extrusion under alkaline conditions conferred a more fibrous microstructure due to the compact gluten network. The alkali addition (sodium carbonate- 0.1-1.0%; sodium bicarbonate- 0.1-1.5%, and sodium hydroxide- 0.1-0.5%) increased the dehydroalanine-derived cross-linking between dehydroalanine and lanthionine and decreased the cystine content leading to a compact and more fibrous structure (180). The resultant wheat gluten extrudates had higher hardness followed by reduced hardness, and higher resilience and chewiness upon increasing alkaline concentration. The decrease in free sulfhydryl content (SH) showed that dehydroalanine-derived cross-linking was less crucial than disulfide cross-linking. Thus, a desired level of alkali is required for improving the textural structures, functional properties (rehydration time and water absorption) and mouthfeel of wheat gluten with more porosity and puffed structure and a higher level of alkali concentration could result in the inhibition of the fibrous structure of gluten extrudate.

Pietsch et al. (181) studied the effect of process conditions (screw and die condition) on wheat gluten polymerization during high moisture extrusion cooking and reported that polymerization conditions were affected by process conditions in the screw section such as temperature at extruder exit and extruder pressure at extruder exit and specific mechanical energy (SME) inputs. The authors noted a significant change in gluten polymerization upon 90-160°C extruder temperature leading to a perceivable anisotropic structure of gluten protein, whereas specific mechanical energy (SME) of inputs and barrel pressure in the investigated range (1.5-3.5 MPa and 32-206 kJ/kg SME) have any significant effect on the wheat gluten polymerization. Screw speed within a limit positively affects the texturization of vegetable proteins. A screw speed of 250-350 rpm was reported to improve the texturization of peanut proteins, whereas increasing the speed further to 450 rpm caused poor texture with a weak fibrous structure (182).

#### 5.1.1 Low moisture extrusion

Low moisture extrusion is commonly used in the food industry with a moisture content of below 30-32% on a wet basis. Low moisture extrusion has a shorter die and a higher temperature (183). Due to the application of high temperatures and shear force, low moisture extrusion has a profound impact on the quality characteristics of the extruded product such as expansion and microstructure (184). By proper selection of processing conditions and feed composition, these quality characteristics can be altered to the desired level. Beck et al. (183) observed increased specific mechanical energy inputs, bulk densities, and air cell densities during low moisture extrusion of pea proteins and pea fiber-fortified rice starch blends as compared to pure rice starch. At higher protein content (42%), a decreased sectional expansion index was recorded due to the distribution of protein and starch in thin layers within the extrudate (183).

#### 5.1.2 High moisture extrusion

High moisture extrusion is widely used for texturizing non-meat proteins for the production of meat analogs (185). High moisture extrusion cooking is characterized by lesser mechanical energy dissipation and higher heat transfer via a larger barrel surface (186). The mass is processed into a dense with nil or minimally expanded strand and immediately cooled down to 100°C prior to its release into the atmosphere. It helps to prevent evaporation of water, expansion, and disruption into small pieces (187). With the help of twin-screw extruders consisting of a long-heated barrel and cooling die channel to cook, form, and solidify the strand, it is possible to prepare mass with moisture content up to 75% during the development of meat analogs (188). In the extruder barrel, two corotating intermeshing screws mix water into this product leading to a paste-like product (186). During this process, heat transfer takes place from barrel walls, and dissipating mechanical energy increase temperatures of 120-160°C with pressure increase in the barrel (188, 189). The elongated barrel improves the retention time of the mass and increases the barrel surface; consequently, improving heat transfer. Upon reaching the mass to die, the mass flows a laminar flow through the cooling channel, cools down, and forms a solid anisotropic structure (187).

High moisture extrusion cooking is commonly used to produce meat analogs by producing desirable texture/fibrous structure to vegetable proteins at a high temperature needed to denature vegetable proteins by the shearing force of screw rotation inside the barrel leading to the unfolding of peptide bonds and the destruction of the three-dimensional structure of proteins resulting in the formation of cross-links of hydrogen, amide, and disulfide bonds between denatured proteins (177).

High moisture extrusion has a long cooling die, facilitating higher texturization, density, elasticity, and retention of nutrients at a lower temperature. The high moisture extrudates had post-processing challenges such as lower aroma, taste, and higher storage cost, thus requiring suitable technological interventions. Protein texturization in dry extrusion (20-40% moisture) caused by superheated vapor and a sudden pressure drop inside the viscoelastic melt facilitates the production of puffing while passing through the die (153, 154) whereas wet extrusion (50-70% moisture) leads to fibrous structure similar to meat.

Wolz and Kulozik (190) reported that by suitable high moisture extrusion processing, protein aggregates of desirable attributes can be formed such as microparticulate in whey proteins extruded with the help of co-rotating twin-screw, aggregate size determined by specific mechanical energy input (SME) which further determined by mass flow and screw speed. The size of aggregates (protein-protein interaction) and micro-particle formation without significant denaturation of proteins during high moisture extrusion is determined by the thermal folding of proteins followed by aggregation (190). Under high moisture extrusion processing of soy, wheat, and pea protein aggregates, disulfide bond formation plays a significant role in determining texture or aggregates (173).

The reaction rate of gluten proteins is affected by barrel temperature, moisture content, and shear force. During low moisture extrusion of wheat gluten, increasing moisture content from 20 to 40% and increasing shear force (mainly for degradation reactions) were observed significantly increase the reaction rate under a closed cavity rheometer (191). With the improvement in the design of extruders and the upgradation of equipment, it became possible to apply some novel and emerging technologies in extrusion, such as supercritical fluid (SCF) extrusion, by utilizing supercritical carbon dioxide gas as a replacement for steam.

A supercritical fluid is a form of dense, compressed gas that has high mass transfer, penetration, and lower viscosity similar to the gas phase at the same time exhibiting solvent power, high density, and decreased surface tension similar to the liquid phase above critical temperature and pressure (13, 14). Supercritical carbon dioxide (SC-CO_2_) application preserved the nutritive value of extrudates, especially heat-labile vitamins and micronutrients, due to its low critical temperature (Tc-31^°^C). Extrudates with better conformation, smoother surface, and uniform cellular structure are obtained by supercritical fluid extrusion due to increased nucleation and decreased gas diffusion (192). Combining the extruder with a 3D printer has great potential in designing and producing plant-based meat analogs with desirable (193).

**FIGURE 2:**
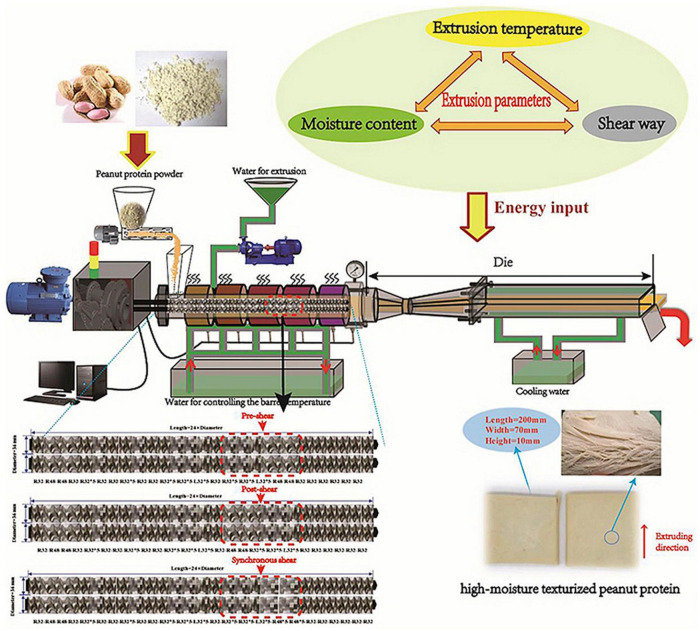
A new insight into the high-moisture extrusion process of peanut protein: from the aspect of the energy input orders and amount [Adapted from Zhang et al. (194)].

The overview of high-moisture extrusion process of peanut protein from the aspect of the energy input orders and amount are presented in Figure 2.

“Reprinted from Journal of Food Engineering, 264, Zhang, J., Liu, L., Jiang, Y., Faisal, S., & Wang, Q., A new insight into the high-moisture extrusion process of peanut protein: From the aspect of the orders and amount of energy input, 109668, Copyright (2020), with permission from Elsevier.”

Various recent processing technologies used for formulating meat analogs are presented in Table 3.

**TABLE 3:** Processing of proteins in formulation and processing of meat analogs.

Meat analogs	Protein source	Processing parameters	Remark	References
**High moisture extrusion**				
Hydrated extrudates	Soy protein isolates and wheat gluten (20, 40, and 60%)	250 rpm screw speed, 140°C die temperature, 50% moisture	● Increasing gluten improved textural attributes with higher fibrous structure with increasing gluten content. ● 40% SPI and 60% wheat gluten had a similar texture to chicken meat.	(195) @A
Extruded meat analogs	65% defatted soy flour, 25% SPI, 10% corn starch, 15 and 30% tuna sawdust		● Tuna sawdust addition softer texture, lower WHC and increased breaking strength, and improved nutritive value. ● Improve antioxidant attributes due to selenium and hydrolysis of peptides.	(178) @A
Fibrous meat substitutes	Pea protein isolates	150 rpm screw speed, 40-140°C feeding to final die temperature, 16 mm screw diameter, 40:60 protein to water feed	● Extrusion did not affect the degree of hydrolysis (3.19-3.50%). ● No new peptide bond formation and amino acid degradation upon high thermal and mechanical energy of moisture extrusion due to no effect on peptide bond formation. ● Formation of covalent disulfide bonding and, to a lesser extent, non-covalent interactions.	(187) @A
Texturized vegetable proteins	SPI (50%), gluten (40%), corn starch (10%), and 15% above mixture replaced by green tea flour	196 rpm screw speed, 150°C die temperature, 47.78% moisture	● Improved nutritive value and antioxidant functionality upon the incorporation of green tea flour. ● Product with similar microstructure and texture to meat.	(196) @A
Extruded soy protein isolates	Soy protein isolates	150-330 rpm screw speed, 140°C die temperature, 40-60% moisture	● Moisture had more impact on extrude quality than screw speed. ● Extrusion at 40% moisture had higher water absorption, color and then extrusion at 50% and 60% moisture	(197) @A
Anisotropic meat analog products	Wheat gluten	180-800 rpm screw speed, 100-155°C barrel temperature, and 10-20 kg/h feed rate	● Polymerization of wheat gluten played a critical role in the formation of anisotropic structures, ● Polymerization increased with increasing thermomechanical treatment resulting in increased hardness	(53) @A
High-moisture meat analogs	Yellow pea -faba bean concentrates and isolates as a replacement to soy protein	400-800 rpm screw speed, 110-150°C -, 60-70% moisture	● The texture of the developed product was affected by the ash, fiber, protein, and water holding capacity of the protein ● Screw speed altered hardness and cutting strength at high moisture (69-70%); a high screw speed and moisture increased texturization	(198) @A
	lupin protein (50% concentrate and 50% isolates)	400-1,800 rpm barrel speed, 138-180°C barrel temperature, 40-68% moisture	● Higher screw speed increased cross-linking and polymerization, leading to higher cutting strength ● Increasing temperature and screw speed along with lower moisture content resulted in denser microstructure and more fiber layers	(185) @A
Pulse protein high moisture meat analogs	Isolates of soy, pea, peanut, and mungbean proteins, wheat gluten	250 rpm screw speed, 100-160^°^C barrel temperature, 50% moisture, 100g/min feed rate	● Meat analogs with high integrity index, low rehydration, and textural attributes ● Meat analogs with isolated soy and isolate pea proteins had a spongy texture ● Increases sulfur-containing amino acids after extrusion in isolated soy protein, isolated pea protein, and wheat gluten	(153) @A
Meat analogs	Soy protein concentrate, 10-40% wheat gluten, 50-80% moisture, natural flavorings	8 zones of temperature ranging from 20-150°C, 30g/min feed rate, 26:1 barrel length to diameter ratio, 4.6:1 screw compression ratio	● High wheat gluten and lower moisture content improved flavor retention of developed products by absorbing volatile flavor compounds ● Glutenin increased plasticity during extrusion, prevented cracks formation, and formed the layered structure of meat analogs	(165) @A
Extruded meat analogs	Soy protein concentrate and wheat gluten	170°C barrel temperature, 57% moisture, feed rate (dry-2.8 kg/h, water-3.6 kg/h)	● Product with 30% wheat gluten had the highest texture score in terms of chewability, hardness, and fibrous structure ● The presence of hydrogen bonds between proteins was noticed from 20, and 30% wheat gluten incorporation, further increase in wheat gluten resulted in higher disulfide bonds	(166) @A
Soy protein meat analog	Soy protein concentrate	50 rpm screw speed, 60°C, 135°C, and 125°C temperature zone, 6 kg/h feed rate, 10 kg/h water feed rate,	● 1.5% iota carrageenan addition form suitable textural and sensory quality meat analogs ● Color of meat analogs not significantly affected by iota carrageenan, increased textural attributes, and reduced expressible moisture and cooking yield	(199) @A
-	50-90% SPC, 10-50% spirulina	600-1,200 rmp screw speed, 57-77% moisture, 140-180°C extrusion temperature	● A firm and fibrous product produced low moisture and high screw speed and temperature with good algal flavor ● Higher spirulina content decreased elasticity, firmness, and fibrousness	(200) @A
Meat analogs	Wheat gluten	1.3-1.5 MPa extruder pressure, 32-206 KJ/Kg SME	● SME did not affect polymerization ● Extruded temperature from 90 to 160°C significantly affected polymerization	(181) @A
Extruded meat analog	SPC (70%) and *Axenochorella protothecoidis* microalgae (30%)	375 rpm screw speed, 44:1 screw length to diameter ratio, 140°C temperature, 60% moisture	● The bright yellow fibrous product had 95% vitamin retention (Vit E and B) content and tenderness ● 50% spray-dried microalgae biomass affected fiber formation, which could be balanced by decreasing moisture content	(201) @A
Insect-based meat analog	15-50% insect *Alphitobius diaperinus* dry biomass, soy fiber, SPC	400 rpm screw speed, 170^°^C temperature, 45% moisture, 3.41 kg/h feed rate	● 5-10% soy fiber incorporation improved texture with more layers ● Hardness, texture, and protein content similar to meat at 40% insect dry biomass and 60% SPC	(202) @A
–	Peanut protein powder (80%), 10% SPI and 10% wheat gluten	210 rpm screw speed, 110°C temp, 55% moisture, 6.0 kg/h feed rate	● SPI and wheat gluten improved the texture ● SPI added to PPP resulted in higher die temperature, lower SME, and denser and smoother product	(203) @A
**Electrospinning, antisolvent precipitation, and mechanical elongation**				
Whole tissue meat analogs	Zein, soy protein	Electrospinning (1-2 kV/cm, distance of collector 20 cm), antisolvent precipitation, and mechanical elongation in water at 40°C for 1 h	● Developed product has attributes similar to tofu-like soy protein isolate gel ● Mechanical elongation produced whole tissue meat analogs textural attributes similar to chicken meat ● Fiber orientation and uniformity were major factors in contributing meaty texture.	(204) @A
-	Whey and SPI mix, soluble SPI and maltodextrin (5:80)	15.5 cm Nozzle-collector distance; 21–25°C temp, 10–15% relative humidity	● Significantly lowered viscosity and increased spinnability for SPI compared to whey protein isolates ● Protein content affects spinnability and fiber appearance	(205) @A
-	SPI, sodium caseinate, whey protein isolates, gelatin in warm water and zein in ethanol as carrier	25 kV voltage, 0.61 mm nozzle diameter, 7.5–15 cm nozzle-collector distance, 3–20 μl/min flow rate	● Gelatin as a carrier medium of food- grade protein to electrospin ● Production of thin fibrils as building blocks	(206) @A
**Couette shear cell/Shear structuring**				
Structured soy-based meat analog	31% soy protein isolates, wheat gluten	90–110°C process temperature, 5–25 min process time, and 5–50 rpm rotation rate	● Couette Cell as a workable and realistic option for producing meat analogs ● Process time and rotation rate are not critical factors in texturization ● Good fibrousness was observed between 90-100°C	(207) @A
Texturized soy meat replacer	Soy protein isolate, wheat gluten at 3.3:1 ratio	120°C process temperature, 7 bar pressure, demi water (69%), slat (1%)	● Designing a 7L capacity Couvette Cell device ● Yield 3 cm thick meat replacer, fiber structured product ● High fibrous structure at 25-35 min process time at 20-30 rpm rotation rate	(208) @A
Meat analog	Soy-protein-wheat gluten, pea protein-wheat gluten	15 min process time at 39/sec, 95-140°C temperature,	● Production of anisotropic fibers ● Air incorporation decreased with increasing temperature ● Pea protein-wheat gluten processed at 140°C had strength similar to soy protein blend ● At 110-120°C, pea protein had similar strength to chicken meat (50-100 kPa)	(209) @A
-	Soy protein (70%) and soy flour (30%)	150°C toasting temp, 30 min hydration, 15 min sheer time, shear temperature: 140°C, 30 rpm shear speed	● Production of good quality viscoelastic fibers with high WHC intermediate NSI for producing meat analogs ● Fibers viscoelastic strength-1-10 kPa	(210) @A

SME-specific mechanical energy, SPI-soy protein isolates, WHC-water holding capacity, NSI-nitrogen solubility index, - no specific terminology.

### 5.2 Spinning

This technology is explored as a novel method for producing ultrathin fibers of vegetable proteins for the development of structured anisotropic meat analogs. The electrospinning process and fiber characteristics are influenced by solution properties such as surface tension, viscosity, and conductivity, and spinnerets operating parameters such as spinneret-collector distance, humidity, and temperature (211). The considerable quantity of waste generation, the requirement of low pH, high salt concentration, and the utilization of chemical additives make this process quite complicated.

Electrospinning of fibers comprises ejecting a polymer solution from a spinneret at high voltage (1-2 kV/cm) toward a collector under electrostatic repulsion. While in the air, its bending becomes unstable and stretches into thin microfibers. Mattice and Marangoni (59) explored the possibility of producing low-cost zein protein-based meat analogs by using electrospinning, antisolvent precipitation, and mechanical elongation technologies. Authors (59, 204) produced ultrathin zein fibers with comparatively lower cost and lower consumption of ethanol by using a 25% (w/w) zein solution in 70:30 ethanol in water loaded in stainless steel spinneret fixed with a 16-gauge syringe at a 0.5 mL/h flow rate by infusion pump operated at 20 kV and maintaining 20 cm distance from collector to spinneret. An instantaneous fibrous network of zein protein was prepared by antisolvent precipitation by incorporating water in excess directly into the ethanol-zein solution. Mechanical elongation of zein fibers was accomplished by dispersing zein in water and incubating at 40^°^C for 1h for network formation.

### 5.3 Couette shear cell

This is a novel technology based on flow-induced structuring/texturization of plant proteins by using a shear cell consisting of a cone-plate rheometer consisting of a stationary top cone and a rotating bottom cone (208). In these Shear Cells, proteins are aligned and produce well-defined fibrous structures upon shear force and heat application. The scalability, batch processing, and variation in shear rate throughout the Shear Cell with increasing distance between radius and cone remain major hurdles in the industrialization of this technology for the production of meat analogs. Couette or concentric cylinder shear cells are used to texturize vegetable proteins by shear bindings, formation of layered structures, and their orientation toward shear flow under the effect of shear-induced concentration fluctuations.

Couette cells (CC) are based on the concept of concentric cylinder rheometers and are used for texturing plant proteins by shear force. Krintiras et al. (208) scaled up the laboratory scale CC with a similar basic principle of viscous fluid flow between two surfaces, moving tangentially relative to one another. The CC consists of 4 main parts viz., an outer stationary cylinder which can be axially displaced with housing and removable lid to access inner half material, an inner rotating cylinder connected to the shaft via a rheodrive unit to control the angular velocity of rotating inner cylinder. The sampling material as plant proteins was placed in the shearing zone, a space between outer and inner cylinders. The processing conditions (pressure, torque response, and specific mechanical energy) are regulated by the rheodrive unit. Steam is used as a heating medium, and a pressure of 7 bar should be maintained throughout the processing. Dekkers et al. (212) developed a fibrous structure of a pectin/soy protein isolate blend under shear-induced structuring. The elongated pectin fibers were oriented toward the direction of shear force, with the length of the fiber depending upon the temperature and pectin concentration.

### 5.4 Additive manufacturing/3D printing

Three-dimensional/3 D printing (3DP) technology, also known as additive manufacturing (AM), rapid manufacturing, rapid prototyping, freedom fabrication, and solid free-form fabrication (SFE), is a novel technology that has vast potential in creating instrumental change in food and agricultural sector (193). It is based on additive manufacturing to develop products from digital Computer-Aided Design (CAD) software. By utilizing this technology, plant proteins can be given desired fibrous and complex muscle-like shapes.

The preparation of high-quality meat analog warrants the utilization of appropriate ingredients through the application of advanced 3D technology to mimic the functional properties of conventional meat without negatively affecting the product features (213). It is possible to develop tailored animal protein-based structures and products with extraordinary flexibility in geometric designs, flavors, and textures, and customized nutrition. Production of 3D printed products requires a reduction in particle size and dilution of flavor, thus reducing the value of premium meat products. Alternatively, this technology could be a better option for utilizing low-value tough meat cuts and meat trimmings (214).

This technology is very energy efficient and sustainable due to the efficient utilization of raw materials with minimum or no waste in the process, easier compositional and nutritional control of the developed product, utilization of novel and exotic plant proteins, saving labor and quick, and supply chain management with transportation by shifting the production facility near to the consumption area (215, 216). For getting the desired efficiency of food bioprinting with high precision, it is required to have proper knowledge of printing parameters and other processing parameters such as rheological attributes, particle size, nozzle characteristics, speed, printing distance, multi-nozzle printers, and post-processing operations such as baking, steaming and frying (193, 215).

During the preparation of meat analogs, protein powder is mixed with water to form a paste, and this paste is structured to a meat-like texture layer by layer by a 3D printer (217). Enzymatic treatment of plant proteins is used to increase cross-link and provide good texture to the product. Recently NovaMeat ^®^ has applied 3D printing technology to develop plant-based analogs of beef steaks, meatballs, burgers and nuggets by using micro-extrusion technology. The developed products had textural and sensory attributes equivalent to conventional products. The Spanish start-up claimed to formulate the biggest cell-based prototype in the world. The company has prepared a hybrid meat analog by mixing mammalian adipose cells with a biocompatible plant-based scaffold.

Shahbazi et al. (213) constructed 3D-printed reduced-fat meat analogs containing a highly porous structure by using reduced-fat soy-based emulsion gels. The authors reported the effect of biosurfactants on the crystalline structure and fibrous sensation of the constructs by reducing the friction coefficients. The reduced-fat meat analogs printed by dodecenyl succinylated inulin and ethyl (hydroxyethyl) cellulose had finer resolution as compared with the product formulated with acetylated and octenyl succinic anhydride modified starches (213).

### 5.5 Freeze structuring/Freeze alignment

The meat-like fibrous texture can be achieved by freezing the plant protein emulsion/slurry subsequently followed by ice-crystal formation. These ice crystals formed help in the development of a well-aligned and unique porous microstructure of plant proteins overlapped in a layered sheet like animal muscles (218, 219). The alignment of protein can be controlled by the proper direction of heat removal, temperature, and rate of freezing. The heat removal from a well-mixed protein solution results in the formation of an isotropic structure. However, if the heat is removed in one direction without mixing, it results in the formation of anisotropic structures. The frozen product is freeze-dried (by utilizing the principle of sublimation) to get the sheet-like structure containing parallel orientation of the proteins. Further, these aligned sheets of proteins are connected by applying a cohesive fibrous product (27, 219).

Plant-protein-based meat analogs similar in texture and sensory attributes to meat were prepared by using freeze alignment (43). The authors (43) achieved successful texturization of a mixture of pea protein and wheat protein (3:1) by (a)- Freezing the protein solution to produce ice crystals that are aligned perpendicular to the cooling surface, (b)- Development of parallel ice crystals zone and entrapping of protein molecules, (c)- Formation of elongated fiber without changing the moisture: protein ratio, (d)- Removal of water/moisture by freeze drying, thus leaving a rigid fibrous mass of protein, (e)- Rehydration of fibrous proteins with an aqueous solution containing fats, color, flavor, and stabilizers. However, the proper control and monitoring of several freezing conditions in this process remain a major hindrance at present (27).

## 6 Prospects and conclusion

The issue of texture and flavor of plant-based meat analogs is quite challenging, and various innovative processing technologies are increasingly being used for texturizing plant proteins similar to the fibrous texture of meat. The typical texture and flavor of the meat results from a very complex process of pH-mediated post-mortem glycolytic and enzymatic changes, comprising numerous compounds and each having its typical role in imparting texture, flavor, and appearance to meat. Further research is needed to explore various protein molecules and their potential role in fabricating the texture and flavor of plant proteins similar to their meat counterparts.

To impart proper meat-like appearance, texture, and sensory attributes, plant proteins are processed into fibrous or fibril texture and added with flavorings, binders, hydrocolloids, colorants, vitamins, and minerals. This high degree of processing and incorporation of a range of ingredients leads to various issues about food safety, permissible limits, clean labeling, efficiency and sustainability, cost, and risk of lack of consumers’ confidence in these products. The processing of plant proteins at higher temperatures and pressure could decrease the nutritive value by degrading heat-labile nutrients and warrants compensatory incorporation of these nutrients as Vit B1, Vit C, flavonoids, phenolics, changing the economic dimension of the production of plant-based meat analogs.

**FIGURE 3:**
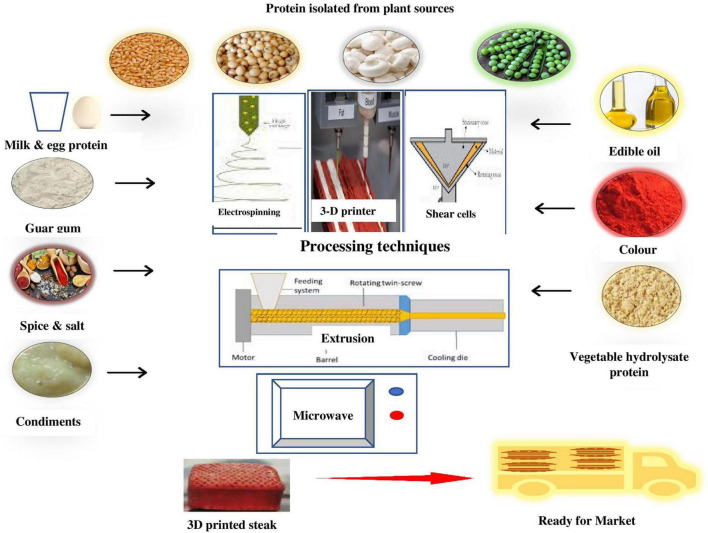
Various aspects of improving the functionality of plant proteins through the development of meat analogs.

The plant-based meat analog sector is exploring, designing innovative technologies (such as electrospinning, three-dimensional/four-dimensional (3D/4D) food printing) and applying already available technologies (such as extrusion, shear pressing, mechanical elongation, and antisolvent extraction) in refining and improving texturizing of plant proteins at a lower environmental cost with suitable combinations of various plant proteins, the application of suitable carbohydrates, hydrocolloids or other non-protein sources. Although these technologies have achieved a significant technological breakthrough in producing fibrous texture and fibrils like meat, their proper popularization and scale-up are needed. Figure 3 represents various aspects of improving the functionality of plant proteins during the development of meat analogs.

Harvesting vast non-conventional protein sources such as oilseed co-products, minor cereals, pseudocereals, microalgae, fungi, and leaves have great potential in ensuring sustainability in the food sector. Leaf protein, oilseed industry co-products, microalgae, and single-cell proteins (SCP) have vast potential and, if harnessed suitably, could solve the issue of sustainability and food security to a great extent in the near future. Improving protein functionality, such as water binding capacity, gelling, and emulsifying properties are also crucial for structuring the plant-based meat analogs.

There is a need to scale up other promising innovative technologies such as 3D/4D food printing, Couvette Shear Cell, and electrospinning. There is a focus on improving the overall process efficiency of the extrusion and fiber formation by lowering energy requirements, minimum or no waste generation, and ease of operations.

## Author contributions

NS, S-JL, and AS: conceptualization. PK, MA, AV, PU, NM, AA, and MH: writing—original draft. UK, NS, S-JL, and AS: writing—review and editing. All authors have read and agreed to the published version of the manuscript.
